# Evidence of antimicrobial resistance-conferring genetic elements among pneumococci isolated prior to 1974

**DOI:** 10.1186/1471-2164-14-500

**Published:** 2013-07-24

**Authors:** Kelly L Wyres, Andries van Tonder, Lotte M Lambertsen, Regine Hakenbeck, Julian Parkhill, Stephen D Bentley, Angela B Brueggemann

**Affiliations:** 1 Department of Zoology, University of Oxford, South Parks Road, Oxford OX1 3PS, United Kingdom; 2 Department of Microbiology Surveillance and Research, Statens Serum Institut, 5 Artillerivej, Copenhagen 2300, Denmark; 3 Department of Microbiology, University Kaiserslautern, Kaiserslautern, Germany; 4 Pathogen Genomics Team, Wellcome Trust Sanger Institute, Wellcome Trust Genome Campus, Hinxton, Cambridge CB10 1SA, United Kingdom

**Keywords:** *Streptococcus pneumoniae*, Antimicrobial resistance, Mobile genetic elements, Tetracycline resistance, ICE elements

## Abstract

**Background:**

Antimicrobial resistance among pneumococci has greatly increased over the past two to three decades. Resistance to tetracycline (*tet*(M)), chloramphenicol (*cat*) and macrolides (*erm*(B) and/or *mef*(A/E)) is generally conferred by acquisition of specific genes that are associated with mobile genetic elements, including those of the Tn*916* and Tn*5252* families. The first tetracycline-, chloramphenicol- and macrolide-resistant pneumococci were detected between 1962 and 1970; however, until now the oldest pneumococcus shown to harbour Tn*916* and/or Tn*5252* was isolated in 1974. In this study the genomes of 38 pneumococci isolated prior to 1974 were probed for the presence of *tet*(M), *cat, erm*(B), *mef*(A/E) and *int* (integrase) to indicate the presence of Tn*916/*Tn*5252*-like elements.

**Results:**

Two Tn*916*-like, *tet*(M)-containing, elements were identified among pneumococci dated 1967 and 1968. The former element was highly similar to that of the PMEN1 multidrug-resistant, globally-distributed pneumococcal reference strain, which was isolated in 1984. The latter element was associated with a streptococcal phage. A third, novel genetic element, designated ICE*Sp*PN1, was identified in the genome of an isolate dated 1972. ICE*Sp*PN1 contained a region of similarity to Tn*5252*, a region of similarity to a pneumococcal pathogenicity island and novel lantibiotic synthesis/export-associated genes.

**Conclusions:**

These data confirm the existence of pneumococcal Tn*916* elements in the first decade within which pneumococcal tetracycline resistance was described. Furthermore, the discovery of ICE*Sp*PN1 demonstrates the dynamic variability of pneumococcal genetic elements and is contrasted with the evidence for Tn*916* stability.

## Background

Pneumococci (*Streptococcus pneumoniae*) are a major bacterial cause of disease for which antimicrobial resistance is of increasing concern. A recent report estimated that in 2009 >24% of disease-causing pneumococci in the USA were tetracycline-nonsusceptible and 8.2% were chloramphenicol-nonsusceptible [1]. In some European countries, such as Spain and France, these rates were higher: 35.1% and 42.3% of pneumococci isolated in these countries, respectively, were tetracycline-nonsusceptible, while 26.1% and 20.3%, respectively were chloramphenicol-nonsusceptible [2]. Similarly, 39.2% of pneumococci in the USA [1], 26.7% of those in Spain and 30.0% of those in France were macrolide-nonsusceptible [2]. In some regions of Asia rates were even higher: 84.8%, 66.8% and 96.4% of pneumococcal isolates were tetracycline-, chloramphenicol- and erythromycin-nonsusceptible, respectively [3].

Among pneumococci, chloramphenicol and tetracycline nonsusceptibility are conferred through acquisition of the *cat* and *tet* genes (most commonly *tet*(M)). The *cat* gene encodes a chloramphenicol acetyltransferase, which catalyses the conversion of chloramphenicol into non-active derivatives. *tet*(M) encodes a ribosomal protection protein shown to initiate reversal of tetracycline-ribosome binding, an event which would otherwise inhibit protein synthesis [4].

Pneumococcal nonsusceptibility to erythromycin and other macrolides is most commonly conferred by acquisition of the *erm*(B) and/or *mef*(A/E) genes [5,6]. *erm*(B) encodes an N-methyltransferase which mediates macrolide-target site alteration [7]. *mef*(A/E) encodes macrolide efflux pumps [8] allowing the resistant cell to reduce its internal macrolide concentration and thereby eliminate the detrimental effects of these drugs.

In recent years it has become apparent that the majority of *tet*(M) and/or *cat* genes among tetracycline- and/or chloramphenicol-nonsusceptible pneumococci are contained within genetic elements known as integrative conjugative elements (ICEs) [9]. *tet*(M) is associated with the Tn*916* family of ICEs, the first of which was described in 1981 from an *Enterococcus faecalis* isolate [10]. *cat* is associated with the Tn*5252* ICE family, first described in 1991 [11], where, besides the typical *int*_Tn*5252*_ integrase, a different integrase (*int*_ICE*Sp*23FST81_) has recently been described [12].

To date, Tn*916*-like ICEs have been identified among >35 bacterial genera [13]. Tn*916* itself is ~18 kb in length and contains 24 genes arranged into functional modules associated with conjugal transfer, recombination, transcriptional regulation and accessory functions (e.g. tetracycline resistance [14]). The recombination module contains the integrase (*int*) and excisionase (*xis*) genes. The *int* gene encodes a tyrosine recombinase responsible for integration and excision of the element from the host chromosome. The product of *xis* drives the directionality of the recombination process, most commonly by promoting excision [14]. Tn*916* has been shown to have the ability to transpose intracellularly and to mediate its own intercellular transfer via conjugation [14]. Successful conjugal transfer between pneumococci and into pneumococci from other species is a strain-dependent property [15,16]; transfer between pneumococci by transformation has also been documented [17]. Aside from Tn*916*, other members of this family may contain alternate accessory genes and/or feature a smaller, independent genetic element integrated within the Tn*916* genetic background, e.g. the *erm*(B)-containing Tn*917* or Omega elements, or the *mef*(E)-containing MEGA (macrolide efflux genetic assembly) element [17-19]*.*

Among pneumococci, Tn*5252*-like ICEs are most commonly identified in association with Tn*916*-like ICEs [9,11,12,18] whereby the resulting composite element is known as Tn*5253* (or Tn*5253*-like) and may be highly variable [9,18]. In addition to the *cat* gene, Tn*5252* (~48 kb in length) contains an independent *int* gene and the *umuDC* gene*,* shown to provide protection from UV damage [20]. In some cases Tn*5252*-like ICEs also contain a lantibiotic synthesis gene cluster [12,18]. Lantibiotics, or lanthionine-containing antibiotics, are antimicrobial compounds with activity against a range of gram-positive bacterial species [21].

There is evidence to suggest that Tn*916*-like and Tn*5252*-like elements were present among pneumococci in the 1970s [22,23] and to our knowledge the oldest pneumococcus for which such an element has been described was isolated in 1974 [11,22]. However, tetracycline-nonsusceptible pneumococci were first identified in 1962 [24], while pneumococcal resistance to erythromycin and chloramphenicol were recognised in 1967 [25] and 1970 [26], respectively. Given the important role of these ICEs in the dissemination of resistance determinants, it therefore seems likely that they, or similar genetic elements, were present among pneumococcal populations from at least as early as the 1960s. The aim of this study was to use our historical genome collection [27] to search for, characterise, and compare such older elements among pneumococci isolated prior to 1974, thus pre-dating the earliest known pneumococcal Tn*916* and *Tn5252* representatives [10,11,22].

## Results

### Nucleotide comparison of pneumococcal ICE int genes

Ten uniquely designated pneumococcal ICEs were identified from Genbank. Four elements (Tn*1131*, Tn*5253*, ICE*Spn*11876 and ICE*Spn*11930) each contained two independent *int* genes. Six elements each contained only a single *int* gene. Alignment of all 14 *int* nucleotide sequences showed four clearly defined clusters; sequences assigned to the same cluster shared >98% nucleotide identity (Table 1).

**Table 1:** Integrase genes identified in specific pneumococcal genetic elements, grouped by nucleotide sequence similarity

**Group**	**Integrase**	**Genetic element**^**a**^	**Genbank**
	**gene type**	**representative**^**a**^	**accession no.**
1	*int*_Tn*916*_	Tn*1311*(a)	FN667862.2
		Tn*1545*	X61025.1
		Tn*2010*	AB426620.1
		Tn*5251*	FJ711160.1
		Tn*5253*(a)	EU351020.1
		Tn*6003*	AM410044.5
		ICE*Spn*11876(a)	FR671404.1
		ICE*Spn*11930(a)	FR671403.1
2	*int*_ICE*Sp*23FST81_	ICE*Spn*11876(b)	FR671404.1
		ICE*Spn*6094	FR670347.2
3	*int*_ICE*Spn*8140_	ICE*Spn*8140	FR671412.1
4	*int*_Tn*5252*_	Tn*1131*(b)	FN667862.2
		Tn*5253*(b)	EU351020.1
		ICE*Spn*11930(b)	FR671403.1

a. (a) and (b) refer to pairs of different integrase genes found in elements Tn*1131*, Tn*5253*, ICE*Spn*11876 and ICE*Spn*11930. Other representatives of genetic elements in these groups may be found in the ICEberg database (http://db-mml.sjtu.edu.cn/ICEberg/).

### Identification of resistance determinants and transposon int genes among pneumococci isolated prior to 1974

In total, 19 unique CCs and 23 unique serotypes/groups were represented by the 38 isolates included in this study (Table 2). The BIGS database BLASTn tool [28] was used to screen the genomes of these isolates for the presence of each of the *tet*(M), *cat*, *erm*(B) and *mef*(A/E) resistance determinants, and one representative of each of the *int* nucleotide sequence clusters. Two isolates, 14/5 (1967) and 18C/3 (1968), were positive for both *int*_Tn*916*_ and *tet*(M). Isolate PN1 (1972) did not possess *tet*(M)*, cat, erm*(B) or *mef*(A/E) resistance determinant genes, although it had *int*_Tn*5252*_. No other isolates were positive for any of the *tet*(M)*, cat, erm*(B) or *mef*(A/E) resistance determinant genes, or any *int* genes.

**Table 2:** Pneumococcal isolates included in this study

**Isolate**	**Serotype**	**Year**	**Country**	**ST**	**CC**	**Genome accession no.**^**b**^
7A/2	7A	1937	Denmark	191	191	ERR025824
14/3	14	1939	Denmark	875	1106	ERR026719
17 F/2	17 F	1939	Denmark	123	113	ERR026724
18C/2	18C	1939	Denmark	4706	113	ERR026186
22A/2	22A	1939	Denmark	7181	490	ERR025822
18B/2	18B	1941	Denmark	4706	113	ERR026193
35C/2	35C	1941	Denmark	7196	113	ERR026192
2/3	2	1943	Denmark	3744	490	ERR026715
35C/3	35C	1943	Denmark	5989	113	ERR025823
23A/2	23A	1945	Denmark	439	439	ERR025821
7B/2	7B	1952	USA	7180	66	ERR025825
7 F/2	7 F	1952	USA	7210	218	ERR026172
9 L/2	9 L	1952	USA	5979	124	ERR026178
9 N/2	9 N	1952	USA	7205	None3782	ERR026179
14/2	14	1952	USA	124	124	ERR025830
17 F/1	17 F	1952	USA	574	574	ERR025831
19 F/8	19 F	1952	Denmark	7229	4399	ERR026190
2/2	2	1956	USA	574	574	ERR025820
10 F/2	10 F	1956	USA	7186	490	ERR025828
11C/1	11C	1957	USA	7201	124	ERR025829
9 N/6	9 N	1960	Denmark	71	66	ERR026180
12 F/3	12 F	1961	Denmark	7228	4399	ERR026195
14/4	14	1961	Denmark	134	124	ERR026188
18 F/1	18 F	1961	USA	7182	490	ERR026173
7 F/3	7 F	1962	Denmark	191	191	ERR026176
9A/1	9A	1962	USA	312	156/162	ERR025826
12 F/2	12 F	1962	USA	7194	Singleton7194	ERR026183
17 F/3	17 F	1962	Denmark	392	392	ERR026725
17 F/4	17 F	1962	Denmark	392	392	ERR026726
19 F/5	19 F	1962	Denmark	7229	4399	ERR026717
19 F/10	19 F	1963	Denmark	7230	Singleton7230	ERR026174
14/5^a^	14	1967	Denmark	15	15	ERR026720
23 F/4	23 F	1967	Australia	7184	Singleton7184	ERR026191
9 V/4	9 V	1968	Denmark	123	113	ERR025827
18C/3^a^	18C	1968	Denmark	7195	113	ERR026716
PN2	4	1969	PNG	7152	Singleton7152	ERR163219
19 F/11	19 F	1972	Denmark	71	66	ERR026175
PN1^a^	6	1972	PNG	7151	277	ERR163220

Note: PNG = Papua New Guinea; ST = Sequence Type; CC = Clonal Complex.

**Figure 1:**
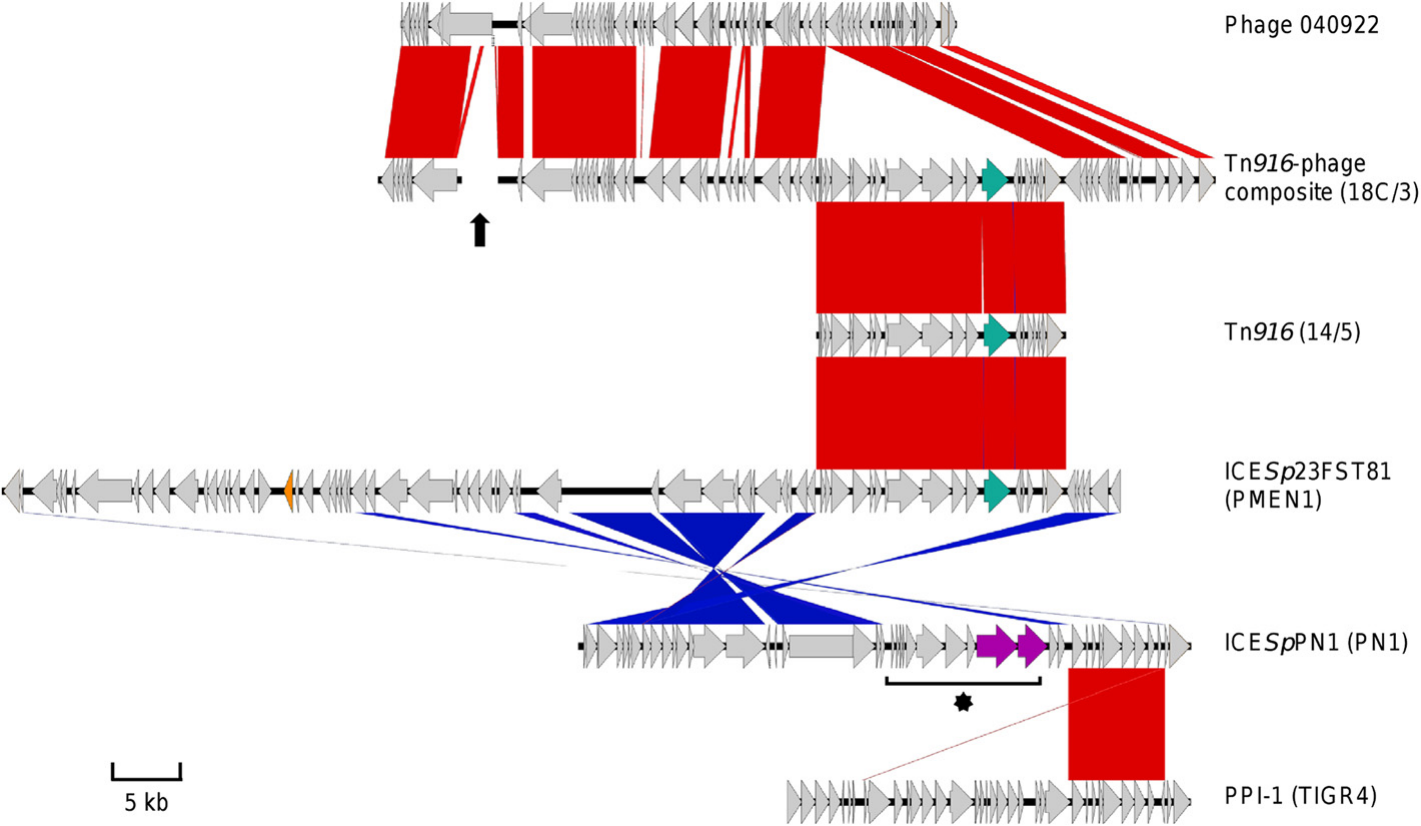
**Comparison of mobile genetic elements identified in this study and those previously described.** Elements are named as in text, and isolates in which these elements were identified are named in parentheses. Predicted genes are depicted by horizontal arrows; cyan arrows represent *tet*(M) resistance genes, the orange arrow indicates *cat*, and purple arrows represent lantibiotic-associated genes. Red bars represent BLASTn matches between sequences. Blue bars represent reverse-oriented BLASTn matches. The region of missing sequence within the Tn*916*-(bacterio)phage composite element is indicated by a vertical black arrow; the asterisk marks an ~11.3 kb insertion in ICE*Sp*PN1 (as described in Results). See Additional file 1 for details about the genes found within the Tn*916*-phage composite and ICE*Sp*PN1 elements.

a. Isolates that possess the mobile elements discussed in this manuscript and are depicted in Figure 1.

b. European Nucleotide Archive accession number.

Within the 14/5 genome, the *int*_Tn*916*_ and *tet*(M) genes were located on a Tn*916* element which was structurally identical (i.e. contained the same putative genes in the same order and was a complete BLASTn match) to the Tn*916* region of ICE*Sp*23FST81 (the PMEN1 pneumococcal reference isolate ICE, Figure 1). In the 14/5 genome this Tn*916* ICE was inserted upstream of the *pspA* locus, which encodes pneumococcal surface protein A.

Within the 18C/3 genome, *int*_Tn*916*_ and *tet*(M) were located on a Tn*916* element, itself associated with a putative bacteriophage showing similarity to the *Streptococcus* phage 040922 (Genbank accession no. FR671406.1; Figure 1) and inserted in the genome downstream of the *trxB* locus, which encodes a pyridine nucleotide-disulphide oxidoreductase. The genome of the 18C/3 bacteriophage contained 48 genes, 31 of which were predicted to encode hypothetical proteins. A further 13 genes were predicted to encode functional phage proteins such as the phage integrase, lytic amidase, holin and structural phage proteins (Additional file 1; Genbank accession no. KC488256). It should be noted here that contiguous sequence could not be obtained across the full length of the 18C/3 Tn*916*-bacteriophage composite element, despite repeated attempts using PCR and conventional Sanger sequencing techniques. The position of the missing region is shown in Figure 1 and likely included part of the *pblB* gene, which may play a role in adhesion [29]. Relative to those of 14/5 and ICE*Sp*23FST81, the 18C/3 Tn*916*-like sequence contained a 155 bp deletion between *orf12* and *tet*(M)*.*

Comparative analyses indicated that PN1 harboured a novel, composite ICE which was inserted in the genome upstream of the *rbgA* locus, which encodes a ribosomal biogenesis GTPase. This novel ICE, designated ICE*Sp*PN1, contained 45 genes and regions of similarity to Tn*5252* and PPI-1 (Pneumococcal Pathogenicity Island 1) as described below (Figure 1 and Additional file 1; Genbank accession no. KC488257).

The 15 5’-most genes of ICE*Sp*PN1 showed similarity to those of the Tn*5252-*like region of ICE*Sp*23FST81 and included *repA*, which encodes replication initiation factor A. A further four and two putative genes also showed similarity to those of Tn*5252* and were separated by a ~11.3 kb insertion (Figure 1). This insertion contained 10 putative genes, including four with ≥96% nucleotide sequence identity to those of a two-component signalling system described for *Streptococcus mitis* strain B6 [30] and *S. pneumoniae* ICE*Spn*8140 [18]. These genes were predicted to encode an ABC-type antimicrobial peptide transporter, an ABC-type transporter permease, a sensor histidine kinase and a response regulator. Directly 3’ of the two-component system cluster were two genes for which the predicted peptide products showed 64% amino acid identity to the LanM lantibiotic and 76% amino acid identity to a lantibiotic transporter, respectively.

The 3’-most ICE*Sp*PN1 gene represented *int*_Tn*5252*_ and was preceded by a putative excisionase gene, predicted to encode a protein with 83% amino acid identity to a putative *Streptococcus sp.* excisionase. Directly upstream of the putative excisionase gene was a region which was structurally identical to part of PPI-1 of TIGR4, another pneumococcal reference isolate (Genbank accession no. NC_003028.3, see Figure 1). This region spanned nine predicted genes, the predicted protein products of which included the PlcR putative transcriptional regulator, an IS200 family transposase, a putative protein kinase, and both a putative ABC-transporter ATP-binding protein and permease.

### Comparison of Tn916 sequence regions

The 14/5 Tn*916-*like nucleotide sequence differed from that of ICE*Sp*23FST81 by only 101 substitutions and a 5 bp insertion within *orf14*. 94 of the nucleotide differences between these sequences were located within the *tet*(M) gene, which is consistent with the previous observation that *tet*(M) possesses a mosaic nucleotide sequence [31]. Additionally, this Tn*916*-like sequence shared >99% nucleotide identity with Tn*916*-like ICEs identified among contemporary bacterial isolates including *Streptococcus suis*, *Streptococcus parauberis* and *Staphylococcus aureus*[32-34]. The 18C/3 Tn*916*-like nucleotide sequence was highly similar to that of the 14/5, differing by only two nucleotide substitutions (and the deletion described above)*.*

## Discussion

This study of an historical collection of pneumococci has led to the discovery of two of the earliest known representatives of pneumococcal Tn*916-*like ICEs and a novel composite ICE. The Tn*916*-like ICEs from pneumococci isolated in 1967 and 1968, respectively, were highly similar to the Tn*916*-like region of ICE*Sp*23FST81 from the PMEN1 reference strain dated 1984 [12], and contemporary Tn*916*-like ICEs. Both of these ICEs contained the tetracycline-resistance determinant *tet*(M), but did not contain any other resistance determinant genes. Identification of such elements among pneumococci isolated in the late 1960s confirms their existence from the first decade within which tetracycline resistance was reported among pneumococci [24]. (Note that tetracycline was released for use in 1948, chloramphenicol in 1947 and erythromycin in 1952 [35].)

The Tn*916*-like element dated 1968 in isolate 18C/3 was inserted within a phage showing similarity to the *Streptococcus* phage 040922 (Genbank accession no. FR671406). Bacteriophages are known to mediate horizontal gene transfer between bacteria through the process of transduction [36], although it is not clear to what extent this process occurs amongst pneumococci, or whether the 040922 phage has retained this ability. Pneumococcal Tn*916-*like ICEs have not generally been associated with phage [9,16,17,19] and no such other phage-associated representatives are present in the Genbank database. Thus it seems unlikely that phage-mediated transduction has played an important role in the dissemination of Tn*916*-like ICEs, although it is possible that the 18C/3 ICE was acquired in this way.

Aside from the *tet*(M) genes described above, no other *tet*(M)*, cat, erm*(B) or *mef*(A/E) resistance determinants were identified among the pneumococcal genomes studied here. There are other resistance mechanisms such as *erm*(A), *erm*(TR) or *tet*(O) that have been found in pneumococci but are believed to be rare [37,38]. We searched for these genes among our genome collection and found no evidence for them, thus in this study we focussed on the common tetracycline, chloramphenicol and erythromycin resistance mechanisms among pneumococci.

In addition to those described above, an ICE-associated *int* gene was identified among one further isolate, PN1, dated 1972. It is also worth noting that this strain was among the earliest penicillin-nonsusceptible pneumococci to be identified, by virtue of the possession of altered penicillin-binding protein genes [27]. Further analyses indicated that this pneumococcus harboured a novel composite ICE, designated ICE*Sp*PN1, which contained regions of similarity to the previously described Tn*5252*-like ICEs and PPI-1 of the TIGR4 reference isolate. Additionally this ICE contained a two-component signalling system gene cluster and two putative lantibiotic biosynthesis-/export- associated genes.

Lantibiotics are small, lanthionine-containing antimicrobial peptides. Synthesis and secretion follow detection of extracellular signals and are auto-regulated by two-component signal systems such as that putatively described above for ICE*Sp*PN1. Antimicrobial activity is achieved following post-translational modification. Production of immunity proteins is required to protect the host cell [21]; however, no such post-translational modification enzyme- or immunity protein- associated genes were identified within ICE*Sp*PN1, calling into question the ability of PN1 to produce a functional version of this lantibiotic.

PPI-1 contains a 5’ region which is highly conserved among pneumococci and a 3’ region which is not conserved (with the exception of the most 3’ gene) [12,39]. ICE*Sp*PN1 contained a region which was structurally identical to the non-conserved region of TIGR4 PPI-1. Previously it was noted that loci within the conserved regions of PPI-1 showed similarity to those of Tn*5252*[39]. Subsequently it was suggested that sequences may be exchanged between these elements via homologous recombination [12]. Such a process could explain the patterns of shared sequence structure described here.

Previous authors have demonstrated the capacity for diversity among pneumococcal ICEs [9,18], and the description of ICE*Sp*PN1 reiterates this dynamic variability. The finding that two pneumococci isolated in the late 1960s harboured Tn*916*-like ICEs, and that these ICEs were highly similar to that of the epidemiologically-successful PMEN1 reference strain and contemporary Tn*916*-like elements, demonstrates the ability of these ICEs to persist, almost unchanged (≥99% nucleotide sequence identity), within bacterial populations for many years. The phenotypic effects associated with possession of such ICEs have an important impact on our ability to treat pneumococcal disease. In this context, understanding the processes driving the spread, maintenance and/or diversification of these elements is of the utmost importance.

## Conclusions

In this study we discovered the oldest known examples of tetracycline resistance-conferring pneumococcal genetic elements: two different Tn*916*-like, *tet*(M)-containing, elements identified among pneumococci dated 1967 and 1968. The former element was highly similar to that of the PMEN1 multidrug-resistant, globally-distributed pneumococcal reference strain isolated in 1984. The latter element was uniquely associated with a streptococcal phage. We also described a novel ICE element in a pneumococcal isolate recovered in Papua New Guinea in 1972, and interestingly, this isolate was also one of the earliest penicillin-nonsusceptible pneumococci. This novel element, designated ICE*Sp*PN1, contained a region of similarity to Tn*5252*, a region of similarity to a pneumococcal pathogenicity island and novel lantibiotic synthesis/export-associated genes. The importance of antimicrobial resistance among pneumococci is unequivocal, and our work sheds further light on how these particular resistance determinants have evolved.

## Methods

### Genomic sequencing, serotype and genotype data

Whole-genome sequence data for pneumococci sampled from an historical isolate collection were previously generated and described [27]. Thirty-six of these genome sequences represented pneumococci isolated prior to 1974 and were thus included in this study (Table 2). The genomes of two additional pneumococci, PN2 and PN1, isolated in 1969 and 1972 respectively (Table 2), had recently been added to our historical isolate collection and were included because they were also isolated before 1974. These genome sequences were generated on the Illumina Hi-seq platform; production of 200 bp insert libraries was followed by 100 nucleotide paired-end sequencing using standard protocols. Illumina reads were assembled to consensus contigs using Velvet [40]. Data were deposited in a BIGS database [28]. Serotype/group and genotype data (as defined by multilocus sequence typing [41]) for all isolates were previously described. Isolates were assigned to clonal complexes (CCs – clusters of genotypes descended from a recent common ancestor) by a modified goeBURST method [27,42].

### Identification of putative ICEs

Reference nucleotide sequences for each of the *tet*(M)*, cat, erm*(B) and *mef*(A/E) resistance determinants were retrieved from Genbank. *int* gene sequences were retrieved from each uniquely designated pneumococcal ICE, identified by interrogation of Genbank using the following search term combinations: Organism = *Streptococcus pneumoniae* + Title = *Transposon*, Organism = *Streptococcus pneumoniae* + Title = Conjug*, Organism = *Streptococcus pneumoniae* + Title = *element*. *int* nucleotide sequences were aligned by MUSCLE [43] and imported to MEGA5 [44] for assignment to clusters (by visual comparison), and sequence identity calculation. The BIGS database BLASTn tool [28] was used to search isolate genomes for the *tet*(M)*, cat, erm*(B) and *mef*(A/E) resistance determinants, and a single representative of each *int* gene cluster.

Isolates positive for any resistance determinant or *int* gene were further studied by extraction of the relevant Velvet contig from the BIGS database and comparison to the genomes of the pneumococcal reference strains R6 and PMEN1 (Genbank accession no. AE007317.1 and NC_011900.1, respectively) using the Artemis Comparison Tool (ACT) [45]. Candidate genetic elements were identified as those which contained one or more of the resistance determinants of interest (i.e. *tet*(M)*, cat, erm*(B) and *mef*(A/E)) and/or *int* genes, and were not similar to any region of the R6 genome, which does not contain any Tn*916*/Tn*5252*-like ICEs. Candidate Tn*916*-like and/or Tn*5252*-like elements were further identified as those which showed similarity to the Tn*916* and/or Tn*5252*-like regions of the PMEN1 reference ICE, ICE*Sp*23FST81 [12]. Regions of the resistant determinant/*int*-containing contigs that did not show similarity to any part of the R6 genome or PMEN1 ICE were extracted and queried against the Genbank database by BLASTn. Sequences representing the best BLAST matches were retrieved from the database for further comparison using ACT.

### Identification of additional ICE regions and genome integration sites

When a single Velvet contig contained putative ICE sequences plus regions showing similarity to the R6 reference genome, the latter were considered to represent the genomic flanking regions of the ICE. Consequently putative ICE genomic integration sites were identified by reference to the R6 genome annotation.

When genomic flanking sequences were not contiguous to the putative ICE sequences, SMALT [46] was used to map Illumina sequence reads to the Velvet consensus assembly. Mapped assemblies were converted to a gap5 database [47]. Illumina reads that mapped to the ends of the putative ICE-containing contigs were checked for the location of their corresponding paired reads. This enabled the identification of consensus assembly contigs representing the adjacent region(s) of the genome. Comparison of these contigs to the R6 genome using ACT identified the putative ICE integration sites. Sequences which were not similar to the R6 genome were considered additional ICE regions and were queried against the Genbank database by BLAST.

Association of the original ICE assembly contigs, additional putative ICE contigs and/or the putative genomic flanking region contigs was confirmed by standard PCR amplification in 25 μl reaction volumes followed by agarose gel electrophoresis (primers available upon request). Conventional Sanger sequencing was used to close sequence gaps within putative ICEs. PCR products to be used for Sanger sequencing were precipitated with 60 μl of 20% PEG (polyethylene glycolate) / 2.5 M NaCl and washed with 70% ETOH. Sequencing was completed as described previously [27].

### Prediction of genes and Tn916 sequence comparison

Putative genes were predicted using Prodigal [48]. Where possible, putative functions were assigned by BLAST match to sequences deposited in Genbank. Tn*916* sequence regions were aligned by MUSCLE [43] and imported to MEGA5 [44] for visual inspection / sequence identity calculation.

## Abbreviations

ABC: ATP-binding cassette; ACT: Artemis Comparison Tool; BIGS: Bacterial Isolate Genome Sequence; cat: Chloramphenicol resistance gene; CC: Clonal complex; erm(B) and/or mef(A/E): Erythromycin resistance genes; ICE: Integrative Conjugative Element; int: Integrase gene; MEGA: Macrolide efflux genetic assembly; MEGA5: Molecular Evolutionary Genetics Analysis 5; PCR: Polymerase chain reaction; PMEN: Pneumococcal Molecular Epidemiology Network; PPI-1: Pneumococcal Pathogenicity Island 1; ST: Sequence type; tet(M): Tetracycline resistance gene; UV: Ultraviolet; xis: Excisionase gene.

## Competing interests

The authors declare that they have no competing interests.

## Authors’ contributions

KLW and ABB designed the study, analysed data, wrote the paper; LML and RH provided bacterial isolates; JP and SDP provided whole genome sequencing data; KLW and AVT assembled whole genome sequence data and performed bioinformatics analyses to close sequence gaps; KLW and ABB performed PCR and Sanger sequencing reactions to manually close sequence gaps. All authors discussed the results, read and approved the manuscript for publication.

## Supplementary Material

Additional file 1: Table S1 (Genes identified in the phage-Tn916 composite element found in pneumococcal isolate 18C/3) and **Table S2.** (Genes identified in the novel ICE element, ICESpPN1, which was found in the pneumococcal isolate PN1). Click here for file

## References

[B1] Jones RN Sader HS Declining antimicrobial susceptibility of *Streptococcus pneumoniae* in the United States: report from the SENTRY antimicrobial surveillance program (1998–2009) Diagn Microbiol Infect Dis 2010 68 334 336 10.1016/j.diagmicrobio.2010.08.024 20955919

[B2] European Antimicrobial Resistance Surveillance Network (EARS-Net) database [http://www.ecdc.europa.eu]

[B3] Kim SH Song J Chung DR Thamlikitkul V Yang Y Wang H Lu M So TM Hsueh PR Yasin RM Changing trends in antimicrobial resistance and serotypes of *Streptococcus pneumoniae* isolates in Asian countries: an Asian Network for Surveillance of Resistant Pathogens (ANSORP) study Antimicrob Agents Chemother 2012 56 3 1418 1426 10.1128/AAC.05658-11 22232285 PMC3294909

[B4] Ambrose K Stephens DS Tuomanen EI Macrolide, quinolone, and other non-β-lactam antibiotic resistance in *Streptococcus pneumoniae* The Pneumococcus 2004 Washington D. C: ASM Press 351 366

[B5] Farrell DJ Klugman KP Pichichero M Increased antimicrobial resistance among nonvaccine serotypes of *Streptococcus pneumoniae* in the pediatric population after the introduction of 7-valent pneumococcal vaccine in the United States Pediatr Infect Dis J 2007 26 2 123 128 10.1097/01.inf.0000253059.84602.c3 17259873

[B6] Calatayud L Ardanuy C Cercenado E Fenoll A Bouza E Pallares R Martin R Liñares J Serotypes, clones, and mechanisms of resistance of erythromycin-resistant *Streptococcus pneumoniae* isolates collected in Spain Antimicrob Agents Chemother 2007 51 9 3240 3246 10.1128/AAC.00157-07 17606677 PMC2043242

[B7] Weisblum B Erythromycin resistance by ribosome modification Antimicrob Agents Chemother 1995 39 3 577 585 10.1128/AAC.39.3.577 7793855 PMC162587

[B8] Tait-Kamradt A Clancy J Cronan M Dib-Hajj F Wondrack L Yuan W Sutcliffe J *mefE* is necessary for the erythromycin-resistant M phenotype in *Streptococcus pneumoniae* Antimicrob Agents Chemother 1997 41 10 2251 2255 9333056 10.1128/aac.41.10.2251 PMC164101

[B9] Henderson-Begg SK Roberts AP Hall LMC Diversity of putative Tn*5253*-like elements in *Streptococcus pneumoniae* Int J Antimicrob Agents 2009 33 4 364 367 10.1016/j.ijantimicag.2008.10.002 19097761

[B10] Franke AE Clewell DB Evidence for a chromosome-borne resistance transposon (Tn916) in *Streptococcus faecalis* that is capable of "conjugal" transfer in the absence of a conjugative plasmid J Bacteriol 1981 145 1 494 502 6257641 10.1128/jb.145.1.494-502.1981 PMC217299

[B11] Ayoubi P Kilic AO Vijayakumar MN Tn5253, the pneumococcal omega (cat tet) BM6001 element, is a composite structure of two conjugative transposons, Tn5251 and Tn5252 J Bacteriol 1991 173 5 1617 1622 1847905 10.1128/jb.173.5.1617-1622.1991 PMC207310

[B12] Croucher NJ Walker D Romero P Lennard N Paterson GK Bason NC Mitchell AM Quail MA Andrew PW Parkhill J Role of conjugative elements in the evolution of the multidrug-resistant pandemic clone *Streptococcus pneumoniae*^Spain23F^ ST81 J Bacteriol 2009 191 5 1480 1489 10.1128/JB.01343-08 19114491 PMC2648205

[B13] Roberts AP Mullany P Tn*916*-like genetic elements: a diverse group of modular mobile elements conferring antibiotic resistance Fems Microbiol Rev 2011 35 5 856 871 10.1111/j.1574-6976.2011.00283.x 21658082

[B14] Roberts AP Mullany P A modular master on the move: the Tn*916* family of mobile genetic elements Trends Microbiol 2009 17 6 251 258 10.1016/j.tim.2009.03.002 19464182

[B15] Celli J Poyart C Trieu-Cuot P Use of an excision reporter plasmid to study the intracellular mobility of the conjugative transposon Tn*916* in gram-positive bacteria Microbiology 1997 143 1253 1261 10.1099/00221287-143-4-1253 9141688

[B16] Santoro F Oggioni MR Pozzi G Iannelli F Nucleotide sequence and functional analysis of the *tet*(M)-carrying conjugative transposon Tn*5251* of *Streptococcus pneumoniae* FEMS Microbiol Lett 2010 308 150 158 20487027 10.1111/j.1574-6968.2010.02002.x

[B17] Del Grosso M Scotto d'Abusco A Iannelli F Pozzi G Pantosti A Tn*2009*, a Tn*916*-like element containing *mef*(E) in *Streptococcus pneumoniae* Antimicrob Agents Chemother 2004 48 6 2037 2042 10.1128/AAC.48.6.2037-2042.2004 15155196 PMC415626

[B18] Croucher NJ Harris SR Fraser C Quail MA Burton J van der Linden M McGee L von Gottberg A Song JH Ko KS Rapid pneumococcal evolution in response to clinical interventions Science 2011 331 6016 430 434 10.1126/science.1198545 21273480 PMC3648787

[B19] Del Grosso M Camilli R Iannelli F Pozzi G Pantosti A The *mef*(E)-carrying genetic element (mega) of *Streptococcus pneumoniae*: Insertion sites and association with other genetic elements Antimicrob Agents Chemother 2006 50 10 3361 3366 10.1128/AAC.00277-06 17005818 PMC1610078

[B20] Munoz-Najar U Vijayakumar MN An operon that confers UV resistance by evoking the SOS mutagenic response in streptococcal conjugative transposon Tn*5252* J Bacteriol 1999 181 9 2782 2788 10217768 10.1128/jb.181.9.2782-2788.1999 PMC93719

[B21] Willey JM van der Donk WA Lantibiotics: peptides of diverse structure and function Annu Rev Microbiol 2007 61 477 501 10.1146/annurev.micro.61.080706.093501 17506681

[B22] Dang-Van A Tiraby G Acar JF Shaw WV Bouanchaud DH Chloramphenicol resistance in *Streptococcus pneumoniae*: enzymatic acetylation and possible plasmid linkage Antimicrob Agents Chemother 1978 13 4 577 583 10.1128/AAC.13.4.577 27138 PMC352291

[B23] Cooksey RC Swenson JM Clark NC Thornsberry C DNA hybridization studies of a nucleotide sequence homologous to transposon Tn*1545* in the "Minnesota" strain of multiresistant *Streptococcus pneumoniae* isolated in 1977 Diagn Microbiol Infect Dis 1989 12 1 13 16 10.1016/0732-8893(89)90038-2 2540933

[B24] Evans W Hansman D Tetracycline-resistant pneumococcus Lancet 1963 281 451 10.1016/s0140-6736(75)91547-0 48665

[B25] Dixon JMS Pneumococcus resistant to erythromycin and lincomycin Lancet 1967 289 573 10.1016/s0140-6736(67)92803-6 4164310

[B26] Cybulska J Jeljaszewicz J Lund E Munksgaard A Prevalence of types of *Diplococcus pneumoniae* and their susceptibility to 30 antibiotics Chemotherapy 1970 15 304 316 10.1159/000220695 4395554

[B27] Wyres KL Lambertsen LM Croucher NJ McGee L von Gottberg A Liñares J JM R Kristinsson KG Beall BW Klugman KP The multidrug-resistant PMEN1 pneumococcus is a paradigm for genetic success Genome Biol 2012 13 11 R103 10.1186/gb-2012-13-11-r103 23158461 PMC3580495

[B28] Jolley KA Maiden MC BIGSdb: Scalable analysis of bacterial genome variation at the population level BMC Bioinformatics 2010 10 11 595 21143983 10.1186/1471-2105-11-595 PMC3004885

[B29] Romero P Croucher NJ Hiller NL Hu FZ Ehrlich GD Bentley SD García E Mitchell TJ Comparative genomic analysis of ten *Streptococcus pneumoniae* temperate bacteriophages J Bacteriol 2009 191 15 4854 4862 10.1128/JB.01272-08 19502408 PMC2715734

[B30] Denapaite D Brückner R Nuhn M Reichmann P Henrich B Maurer P Schähle Y Selbmann P Zimmermann W Wambutt R The genome of *Streptococcus mitis* B6 - what is a commensal? PLoS One 2010 5 2 e9426 10.1371/journal.pone.0009426 20195536 PMC2828477

[B31] Oggioni MR Dowson CG Smith JM Provvedi R Pozzi G The tetracycline resistance gene *tet*(M) exhibits mosaic structure Plasmid 1996 35 3 156 163 10.1006/plas.1996.0018 8812782

[B32] Holden MTG Hauser H Sanders M Ngo TH Cherevach I Cronin A Goodhead I Mungall K Quail MA Price C Rapid evolution of virulence and drug resistance in the emerging zoonotic pathogen *Streptococcus suis* PLoS ONE 2009 4 7 e6072 10.1371/journal.pone.0006072 19603075 PMC2705793

[B33] Meng F Kanai K Yoshikoshi K Structural characterization of Tn*916*-like element in *Streptococcus parauberis* serotype II strains isolated from diseased Japanese flounder Lett Appl Microbiol 2009 48 6 770 776 19344360 10.1111/j.1472-765X.2009.02609.x

[B34] Schijffelen MJ Boel CH van Strijp JA Fluit AC Whole genome analysis of a livestock-associated methicillin-resistant *Staphylococcus aureus* ST398 isolate from a case of human endocarditis BMC Genomics 2010 11 376 10.1186/1471-2164-11-376 20546576 PMC2900268

[B35] Palumbi SR Humans as the world's greatest evolutionary force Science 2001 293 1786 1790 10.1126/science.293.5536.1786 11546863

[B36] Brüssow H Canchaya C Hardt W-D Phages and the evolution of bacterial pathogens: from genomic rearrangements to lysogenic conversion Microbiol Mol Biol Rev 2004 68 3 560 602 10.1128/MMBR.68.3.560-602.2004 15353570 PMC515249

[B37] Luna VA Roberts MC The presence of the *tetO* gene in a variety of tetracycline-resistant *Streptococcus pneumoniae* serotypes from Washington State J Antimicrob Chemother 1998 42 5 613 619 10.1093/jac/42.5.613 9848445

[B38] Lambertsen LM Ekelund K Hansen DS Kaltoft M Christensen JJ Hammerum AM Erythromycin resistance caused by *erm(A)* subclass *erm(TR)* in a Danish invasive pneumococcal isolate: are *erm(A)* pneumococcal isolates overlooked? Scand J Infect Dis 2008 40 6 7 10.1080/00365540701854717 18584554

[B39] Brown JS Gilliland SM Holden DW A *Streptococcus pneumoniae* pathogenicity island encoding an ABC transporter involved in iron uptake and virulence Mol Microbiol 2001 40 3 572 585 10.1046/j.1365-2958.2001.02414.x 11359564

[B40] Zerbino DR Birney E Velvet: algorithms for *de novo* short read assembly using de Bruijn graphs Genome Res 2008 18 821 829 10.1101/gr.074492.107 18349386 PMC2336801

[B41] Enright MC Spratt BG A multilocus sequence typing scheme for *Streptococcus pneumoniae*: identification of clones associated with serious invasive disease Microbiology 1998 144 3049 3060 10.1099/00221287-144-11-3049 9846740

[B42] Francisco AP Bugalho M Ramirez M Carriço JA Global optimal eBURST analysis of multilocus typing data using a graphic matroid approach BMC Bioinformatics 2009 10 152 10.1186/1471-2105-10-152 19450271 PMC2705362

[B43] Edgar RC MUSCLE: multiple sequence alignment with high accuracy and high throughput Nucleic Acids Res 2004 32 5 1792 1797 10.1093/nar/gkh340 15034147 PMC390337

[B44] Tamura K Peterson D Peterson N Stecher G Nei M Kumar S MEGA5: Molecular Evolutionary Genetics Analysis using maximum likelihood, evolutionary distance, and maximum parsimony methods Mol Biol Evol 2011 28 10 2731 2739 10.1093/molbev/msr121 21546353 PMC3203626

[B45] Carver TJ Rutherford KM Berriman M MA R Barrell BG Parkhill J ACT: the Artemis comparison tool Bioinformatics 2005 21 16 3422 3423 10.1093/bioinformatics/bti553 15976072

[B46] Wellcome Trust Sanger Institute - SMALT [http://www.sanger.ac.uk/resources/software/smalt/]

[B47] Bonfield JK Whitwham A Gap5–editing the billion fragment sequence assembly Bioinformatics 2010 26 14 1699 1703 10.1093/bioinformatics/btq268 20513662 PMC2894512

[B48] Hyatt D Chen GL LoCascio PF Land ML Larimer FW Hauser LJ Prodigal: prokaryotic gene recognition and translation initiation site identification BMC Bioinformatics 2010 11 119 10.1186/1471-2105-11-119 20211023 PMC2848648

