# Correction: Evidence of antimicrobial resistance-conferring genetic elements among pneumococci isolated prior to 1974

**DOI:** 10.1186/s12864-023-09775-7

**Published:** 2023-11-03

**Authors:** Kelly L. Wyres, Andries van Tonder, Lotte M. Lambertsen, Regine Hakenbeck, Julian Parkhill, Stephen D. Bentley, Angela B. Brueggemann

**Affiliations:** 1 Department of Zoology, University of Oxford, South Parks Road, Oxford, OX1 3PS United Kingdom; 2 Department of Microbiology Surveillance and Research, Statens Serum Institut, 5 Artillerivej, 2300 Copenhagen, Denmark; 3 Department of Microbiology, University Kaiserslautern, Kaiserslautern, Germany; 4 Pathogen Genomics Team, Wellcome Trust Sanger Institute, Wellcome Trust Genome Campus, Hinxton, Cambridge, CB10 1SA United Kingdom

**Correction: BMC Genomics 14, 500 (2013)**

**https://doi.org/10.1186/1471-2164-14-500**

Following publication of the original article [1], the authors identified a typographical error in Table 2 related to the accession numbers listed for genomes PN1 and PN2. The correct accession numbers are PN1, ERR163219 and PN2, ERR163220. The genomic analyses as described in the article were performed on the correct genomes, and the assembled genomes were correctly made public in PubMLST: PN1, PubMLST id 25638 and PN2, PubMLST id 37770. The authors apologise for any confusion caused by the typographical errors in Table 2.

**Table 2:** Pneumococcal isolates included in this study

Isolate	Serotype	Year	Country	ST	CC	Genome accession no.^b^
7A/2	7A	1937	Denmark	191	191	ERR025824
14/3	14	1939	Denmark	875	1106	ERR026719
17 F/2	17 F	1939	Denmark	123	113	ERR026724
18C/2	18C	1939	Denmark	4706	113	ERR026186
22A/2	22A	1939	Denmark	7181	490	ERR025822
18B/2	18B	1941	Denmark	4706	113	ERR026193
35C/2	35C	1941	Denmark	7196	113	ERR026192
2/3	2	1943	Denmark	3744	490	ERR026715
35C/3	35C	1943	Denmark	5989	113	ERR025823
23A/2	23A	1945	Denmark	439	439	ERR025821
7B/2	7B	1952	USA	7180	66	ERR025825
7 F/2	7 F	1952	USA	7210	218	ERR026172
9 L/2	9 L	1952	USA	5979	124	ERR026178
9 N/2	9 N	1952	USA	7205	None3782	ERR026179
14/2	14	1952	USA	124	124	ERR025830
17 F/1	17 F	1952	USA	574	574	ERR025831
19 F/8	19 F	1952	Denmark	7229	4399	ERR026190
2/2	2	1956	USA	574	574	ERR025820
10 F/2	10 F	1956	USA	7186	490	ERR025828
11C/1	11C	1957	USA	7201	124	ERR025829
9 N/6	9 N	1960	Denmark	71	66	ERR026180
12 F/3	12 F	1961	Denmark	7228	4399	ERR026195
14/4	14	1961	Denmark	134	124	ERR026188
18 F/1	18 F	1961	USA	7182	490	ERR026173
7 F/3	7 F	1962	Denmark	191	191	ERR026176
9A/1	9A	1962	USA	312	156/162	ERR025826
12 F/2	12 F	1962	USA	7194	Singleton7194	ERR026183
17 F/3	17 F	1962	Denmark	392	392	ERR026725
17 F/4	17 F	1962	Denmark	392	392	ERR026726
19 F/5	19 F	1962	Denmark	7229	4399	ERR026717
19 F/10	19 F	1963	Denmark	7230	Singleton7230	ERR026174
14/5^a^	14	1967	Denmark	15	15	ERR026720
23 F/4	23 F	1967	Australia	7184	Singleton7184	ERR026191
9 V/4	9 V	1968	Denmark	123	113	ERR025827
18C/3^a^	18C	1968	Denmark	7195	113	ERR026716
PN2	4	1969	PNG	7152	Singleton7152	**ERR163220**
19 F/11	19 F	1972	Denmark	71	66	ERR026175
PN1^a^	6	1972	PNG	7151	277	**ERR163219**

*PNG* Papua New Guinea, *ST* Sequence Type, *CC* Clonal Complex

^a^ Isolates that possess the mobile elements discussed in this manuscript and are depicted in Figure 1

^b.^European Nucleotide Archive accession number

The corrected Table 2 is supplied in this correction article with the corrections marked in bold typeface.
