# Supplementary material for: Evidence of antimicrobial resistance-conferring genetic elements among pneumococci isolated prior to 1974
Source: BMC Genomics. 2013 Jul 24;14:500. doi: 10.1186/1471-2164-14-500 (PMC3726389; doi:10.1186/1471-2164-14-500)
Supplement: Additional file 1: Table S1 — (Genes identified in the phage-Tn916 composite element found in pneumococcal isolate 18C/3) and Table S2. (Genes identified in the novel ICE element, ICESpPN1, which was found in the pneumococcal isolate PN1). [file 1471-2164-14-500-S1.pdf]

Additional file 1 for "Evidence of antimicrobial resistance-conferring genetic elements among pneumococci isolated prior to 1974"

Kelly L. Wyres, Andries van Tonder, Lotte M Lambertsen, Regine Hakenbeck, Julian Parkhill, Stephen D. Bentley and Angela B. Brueggemann

Supplementary Table 1.

Genes identified in the phage-Tn*916* composite element found in pneumococcal isolate 18C/3.

Gene[a]	O[b]	Bp[c]	Predicted Product
ICESp18C_3_00001	R	957	phage lytic amidase
ICESp18C_3_00002	R	336	phage holin, LL-H family
ICESp18C_3_00003	R	300	hypothetical protein
ICESp18C_3_00004	R	351	hypothetical protein
ICESp18C_3_00005	R	204	conserved domain protein
ICESp18C_3_00006	R	117	hypothetical protein
ICESp18C_3_00007	R	unk[d]	--
ICESp18C_3_00008	R	351	prophage protein
ICESp18C_3_00009	R	3606	TMP repeat family
ICESp18C_3_00010	R	351	hypothetical protein
ICESp18C_3_00011	R	381	prophage protein
ICESp18C_3_00012	R	414	putative major tail protein
ICESp18C_3_00013	R	369	putative conserved structural protein
ICESp18C_3_00014	R	327	hypothetical protein
ICESp18C_3_00015	R	339	hypothetical protein
ICESp18C_3_00016	R	312	hypothetical protein
ICESp18C_3_00017	R	189	hypothetical protein
ICESp18C_3_00018	R	183	hypothetical protein
ICESp18C_3_00019	R	846	major phage head protein
ICESp18C_3_00020	R	585	phage scaffold protein
ICESp18C_3_00021	R	1349	putative phage head morphogenesis protein
ICESp18C_3_00022	R	1302	phage portal
ICESp18C_3_00023	R	1299	phage terminase large subunit
ICESp18C_3_00024	R	501	phage terminase small subunit
ICESp18C_3_00025	R	405	phage protein
ICESp18C_3_00026	R	438	hypothetical protein
ICESp18C_3_00027	R	459	hypothetical protein
ICESp18C_3_00028	R	162	hypothetical protein
ICESp18C_3_00029	R	696	hypothetical protein
ICESp18C_3_00030	R	1344	hypothetical protein
ICESp18C_3_00031	R	822	hypothetical protein
ICESp18C_3_00032	R	507	hypothetical protein
ICESp18C_3_00033	R	828	hypothetical protein
ICESp18C_3_00034	R	318	hypothetical protein
ICESp18C_3_00035[e]	F	120	hypothetical protein
ICESp18C_3_00036[e]	F	315	conjugative transposon protein
ICESp18C_3_00037[e]	F	384	conjugative transposon protein
ICESp18C_3_00038[e]	F	1386	conjugative transposon FtsK/SpoIIIE-family protein

ICESp18C_3_00039[e]	F	186	conjugative transposon FtsK/SpoIIIE-family
ICESp18C_3_00040[e]	F	1206	putative conjugative transposon replication initiation factor
ICESp18C_3_00041[e]	F	222	conjugative transposon protein
ICESp18C_3_00042[e]	F	498	conjugative transposon protein
ICESp18C_3_00043[e]	F	231	putative conjugative transposon membrane protein
ICESp18C_3_00044[e]	F	2448	conjugative transposon ATP/GTP-binding protein
ICESp18C_3_00045[e]	F	2178	conjugative transposon membrane protein
ICESp18C_3_00046[e]	F	1002	putative cell wall hydrolase
ICESp18C_3_00047[e]	F	915	putative conjugative transposon exported protein
tet(M)[e]	F	1920	conjugative transposon tetracycline resistance protein, tet(M)
ICESp18C_3_00049[e]	R	354	putative conjugative transposon regulatory protein
ICESp18C_3_00050[e]	F	147	hypothetical protein
ICESp18C_3_00051[e]	F	423	putative conjugative transposon regulatory protein
ICESp18C_3_00052[e]	F	231	hypothetical protein
ICESp18C_3_00053[e]	R	129	hypothetical protein
ICESp18C_3_00054[e]	R	252	hypothetical protein
xis[e]	F	204	excisionase
int[e]	F	1218	integrase
ICESp18C_3_00057	R	1203	hypothetical protein
ICESp18C_3_00058	R	486	hypothetical protein
ICESp18C_3_00059	R	360	hypothetical protein
ICESp18C_3_00060	R	483	hypothetical protein
ICESp18C_3_00061	R	765	hypothetical protein
ICESp18C_3_00062	R	231	hypothetical protein
ICESp18C_3_00063	R	342	hypothetical protein
ICESp18C_3_00064	F	240	hypothetical protein
ICESp18C_3_00065	R	171	hypothetical protein
ICESp18C_3_00066	R	204	hypothetical protein
ICESp18C_3_00067	F	690	hypothetical protein
ICESp18C_3_00068	F	792	hypothetical protein
ICESp18C_3_00069	F	951	hypothetical protein
int	F	1128	phage integrase

a. Genes listed in order from left to right as depicted in Figure 1.
b. Orientation of the gene: F, forward; R, reverse.
c. Bp: gene length in base pairs.
d. Precise length of the sequence gap is unknown.
e Tn*916*-associated region.

Supplementary Table 2.

Genes identified in the novel ICE element, ICE*Sp*PN1, which was found in the pneumococcal isolate PN1.

Gene[a]	O[b]	Bp[c]	Predicted Product
PN1_ICE_00001	F	174	hypothetical protein
repA	F	780	replication initiation protein A, RepA
PN1_ICE_00003	F	102	hypothetical protein
PN1_ICE_00004	F	1359	putative DNA methylase
PN1_ICE_00005	F	450	hypothetical protein
PN1_ICE_00006	F	381	arsenate reductase-like glutaredoxin family protein
PN1_ICE_00007	F	234	hypothetical protein
PN1_ICE_00008	F	663	putative caax amino protease family protein
PN1_ICE_00009	F	591	hypothetical protein
PN1_ICE_00010	F	810	hypothetical protein
PN1_ICE_00011	F	729	truncated traD/traG family protein
PN1_ICE_00012	F	243	hypothetical protein
PN1_ICE_00013	F	855	putative membrane protein
PN1_ICE_00014	F	360	hypothetical protein
PN1_ICE_00015	F	2358	hypothetical protein
PN1_ICE_00016	F	2814	M23 peptidase domain protein
PN1_ICE_00017	R	609	hypothetical protein pseudogene
PN1_ICE_00018	R	165	hypothetical protein
PN1_ICE_00019	F	411	Ig domain protein, 2 domain protein
PN1_ICE_00020	F	6225	putative conjugative transposon DNA recombination protein pseudogene
PN1_ICE_00021	F	285	hypothetical protein
PN1_ICE_00022	F	300	hypothetical protein
PN1_ICE_00023	F	183	hypothetical protein
PN1_ICE_00024	F	183	hypothetical protein
PN1_ICE_00025	F	177	hypothetical protein
PN1_ICE_00026	F	108	hypothetical protein
PN1_ICE_00027	F	738	ABC-type antimicrobial peptide transport protein
PN1_ICE_00028	F	2016	ABC transporter permease
PN1_ICE_00029	F	1575	histidine kinase
PN1_ICE_00030	F	597	response regulator
PN1_ICE_00031	F	2955	putative lantibiotic biosynthesis protein
PN1_ICE_00032	F	2124	putative lantibiotic transporter protein
PN1_ICE_00033	F	642	putative parvulin-like peptidyl-prolyl isomerase
PN1_ICE_00034	F	498	hypothetical protein
plcR	F	867	putative transcriptional regulator, PlcR
PN1_ICE_00036	F	135	hypothetical protein
PN1_ICE_00037	F	252	hypothetical protein
PN1_ICE_00038	F	303	hypothetical protein
PN1_ICE_00039	F	1347	putative protein kinase
PN1_ICE_00040	F	918	ABC transporter, ATP-binding protein
PN1_ICE_00041	F	801	putative ABC-2 transporter permease protein
PN1_ICE_00042	F	474	transposase, IS200 family
PN1_ICE_00043	F	222	hypothetical protein
PN1_ICE_00044	F	270	putative excisionase

PN1_ICE_00045	F	1509	putative integrase

a. Genes listed in order from left to right as depicted in Figure 1.
b. Orientation of the gene: F, forward; R, reverse.
c. Bp: gene length in base pairs.
